# Physical Hybrid of Nanographene/Carbon Nanotubes as Reinforcing Agents of NR-Based Rubber Foam

**DOI:** 10.3390/polym13142346

**Published:** 2021-07-17

**Authors:** Sahar Shojaie, Ali Vahidifar, Ghasem Naderi, Elham Shokri, Tizazu H. Mekonnen, Elnaz Esmizadeh

**Affiliations:** 1 Department of Polymer Science and Engineering, Faculty of Engineering, University of Bonab, Bonab P.O. Box 5551761167, Iran; Sahar.Shojaeiiii318075@gmail.com (S.S.); Ali.Vahidifar@airboss.com (A.V.); 2 Department of Polymer Processing, Iran Polymer and Petrochemical Institute, Tehran P.O. Box 1497713115, Iran; G.Naderi@ippi.ac.ir; 3 Department of Chemical Engineering, Faculty of Engineering, University of Bonab, Bonab P.O. Box 5551761167, Iran; Elham.Shokri@bonabu.ac.ir; 4 Department of Chemical Engineering, Faculty of Engineering, University of Waterloo, Waterloo, ON N2L 3G1, Canada; Tizazu.Mekonnen@uwaterloo.ca; 5 Construction Research Center, National Research Council Canada, 1200 Montreal Rd., Ottawa, ON K1A 0R6, Canada

**Keywords:** rubber foam, polymer nanocomposites, mechanical response, hybrid nanofillers, natural rubber

## Abstract

Natural rubber (NR) foams reinforced by a physical hybrid of nanographene/carbon nanotubes were fabricated using a two-roll mill and compression molding process. The effects of nanographene (GNS) and carbon nanotubes (CNT) were investigated on the curing behavior, foam morphology, and mechanical and thermal properties of the NR nanocomposite foams. Microscope investigations showed that the GNS/CNT hybrid fillers acted as nucleation agents and increased the cell density and decreased the cell size and wall thickness. Simultaneously, the cell size distribution became narrower, containing more uniform multiple closed-cell pores. The rheometric results showed that the GNS/CNT hybrids accelerated the curing process and decreased the scorch time from 6.81 to 5.08 min and the curing time from 14.3 to 11.12 min. Other results showed that the GNS/CNT hybrid improved the foam’s curing behavior. The degradation temperature of the nanocomposites at 5 wt.% and 50 wt.% weight loss increased from 407 °C to 414 °C and from 339 °C to 346 °C, respectively, and the residual ash increased from 5.7 wt.% to 12.23 wt.% with increasing hybrid nanofiller content. As the amount of the GNS/CNT hybrids increased in the rubber matrix, the modulus also increased, and the T_g_ increased slightly from −45.77 °C to −38.69 °C. The mechanical properties of the NR nanocomposite foams, including the hardness, resilience, and compression, were also improved by incorporating GNS/CNT hybrid fillers. Overall, the incorporation of the nano hybrid fillers elevated the desirable properties of the rubber foam.

## 1. Introduction

In recent decades, polymer nanocomposites have received a great deal of attention, since adding only a small fraction of nanofillers to the polymer matrix significantly improves their properties [1]. This concept has been successfully applied to thermoplastics [2], elastomers and thermoset elastomers [3]. Today, a wide array of different nanofillers are available, such as nanoclay [4], carbon nanotubes (CNTs) [5], graphene nanosheets (GNSs) [6,7], nano cellulose [8], and halloysite nanotubes [9], which are frequently used in polymer matrices as reinforcing agents. CNTs show many desirable properties related to flexibility, high strength, ability to withstand transverse and torsional displacements, tensile strength and compressive strength without breaking [10]. GNSs are recognized as one of the most effective nanomaterials because of their high potential for mechanical-property improvement due to the combination of enormous specific surface area, strong filler–matrix adhesion, and the extraordinary mechanical properties of the sp2 carbon bonding network [11]. Despite having numerous extraordinary properties, the commercialization of these two carbon nanomaterial allotropes is still limited due to their tendency to agglomerate (bundle and layer forming behavior for CNT and GNS, respectively) [12].

Rubber foam is an interesting material, as it can be used in a diverse range of applications, such as insulation, packaging, and cushioning. The foam can be fabricated according to specific requirements using different types of polymers and blowing agents. A foam’s properties are controlled by its density, cell structure, and the chemistry of the raw materials used [13]. Rubber foams can be classified into two groups based on their cell structure: open-cell foams [14,15,16], where the gas phases (cells) are linked to each other, and closed-cell foams, in which the cells have isolated or separated cell walls [17]. The incorporation of various types of nanofiller has been a commonly used measure to improve the mechanical, thermal, and electrical properties of rubber foams [18]. However, there are still challenges in achieving a segregated filler network wherein the nanofillers are arranged to produce a 3-D network, which percolates the polymer volume. Recently, the combined use of nanofillers as a physical and chemical hybrid has exhibited a synergistic effect that led to outstanding enhanced performance of nanocomposites with respect to employing only one type of reinforcement [19,20,21]. Since the building blocks of a nanocomposite are of nanoscale, many interfaces exist between the two intermixed solid reinforcement phases and the superior properties arise from phase interactions at these interfaces. Ponnamma et al. showed that morphology of NR-CNT composite gradually evolves from a discontinuous set of aggregated clusters to a continuous 3-D network upon the replacement of CNT by nanographene by 50 percent (50:50 hybrid nanofiller) [22]. King et al. suggested that the synergism of CNT and GNS is derived from two main mechanisms: CNTs act as bridges between GNSs and GNSs fill the space between CNTs [23]. Shim et al. presented a predictive model to quantitatively explain the synergistic behavior of CNT/GNS hybrid, which was achieved by featuring an increased number of contacts between GNSs and CNTs [24].

The knowledge of how to control the properties of rubber foams reinforced by hybrid nanofillers is of fundamental importance in order to determine the final performance and application of the product. Although several studies on NR solid nanocomposites reinforced by hybrid nanomaterial are available [22], the literature still lacks information on their foams. Thus, the novelty of this work lies in its primary objective, which is to assess the fabrication of NR nanocomposite foams reinforced by a CNT/GNS hybrid. The present article intends to address the gap by studying how various CNT/GNS physical hybrid loadings influence the curing behavior, the foam morphology, and the mechanical and thermal properties of NR nanocomposite foams.

## 2. Materials and Methods

Standard Malaysian NR (SMR) with a trade name of SMR-20 (Mooney viscosity of ML (1 + 4) @100 °C) was obtained from the Malaysian Rubber Company, Sungai Lalang, Malaysia. Non-functionalized (pristine) carbon nanotubes (CNTs) with an outer diameter of 8–15 nm (95% < purity) was purchased from Notrino, Iran. C-750 graphene nanosheets (GNSs) with a surface area of 750 m^2^/g and an average thickness of 1–5 nm were obtained from XG Science (Lansing, MI, USA). Sulfur, Carbon black, DMF, paraffin oil, MBTS(2-2’-Dithiobis benzothiazole), CBS (N-cyclohexyl-2-benzothiazolesulfenamide), stearic acid, and ZnO were commercial grades. The chemical blowing agent azodicarbonamide (ADC) was provided by the Letai Chemical Plant (Beijing, China).

Before compounding, CNTs and GNSs were kept in an oven at 90 °C for 15 h to eliminate trace moisture. We dispersed 3.5 g of the dried CNTs and GNSs in 200 mL DMF (solvent) in a separate beaker. Each sample was exposed to an ultrasonic process (400 W) in three 5-min steps to improve the dispersion quality. 

An open two-roll mill was then used for compounding the formulation at an ambient temperature. For this, 20 g of NR was masticated in a two-roll mill for 1 min and dissolved in 200 cc of DMF under stirring. The pre-prepared CNTs/DMF solution was then added to half of the NR solution, and the GNSs/DMF solution was added to the other half of the NR solution and placed separately in an ultrasonic bath for two 30-min steps. The two dispersions were then mixed and placed in the same ultrasonic bath for 5 min. To obtain a masterbatch, the samples were dried in a vacuum oven at 70 °C until all DMF was removed and the samples’ weight remained unchanged. Table 1 shows the compounding formulations. First, 57 g NR was masticated in a two-roll mill for 5 min, and after sufficient mastication, the masterbatch was carefully added, and the compounds were milled for 3 min. Other compounding ingredients, except for the curing agent, were then fed gradually into the mill, and the compounding continued for 2 min. In the end, the curing agent was added, and the compounding continued for another 2 min.

The cure behavior of the compounds was determined using an Oscillating Disk Rheometer (ODR) model 4308 (Zwick Co., Ulm, Germany) at a temperature of 160 °C, according to ASTM D22044. An optical microscope was employed to study the foam morphology, cell size, and cell size distribution. Resilience was measured at a temperature of 25 °C using a resilience tester (Frank GMBH, Birkenau, Germany), in accordance with ASTM D1054. A Shore-A type hardness test was performed at room temperature to measure the stiffness of the foams by a hardness tester (Zwick Co. 3100, Germany) according to ASTM D2240. To study the nanofiller’s effect on the NR foams’ mechanical properties, a compression test was performed using (Hiwa-400, Urmia, Iran) according to ASTM D412. Thermogravimetric analysis (TGA) was carried out to estimate the thermal stability of the foams using a Pyris TGA (Perkin-Elmer, Waltham, MA, USA) in the temperature range of 25 °C to 1000 °C under an oxygen atmosphere at a heating rate of 10 °C/min. To study the dynamical mechanical properties of samples, DMTA test was carried out using (DMA 2000 TRITEC, Edinburgh, England). The test was performed by applying a bending force with a frequency of 4 Hz at a heating rate of 10 °C/min in the temperature range of −100 °C to 100 °C.

## 3. Results and Discussion

### 3.1. Curing Behavior

**Figure 1:**
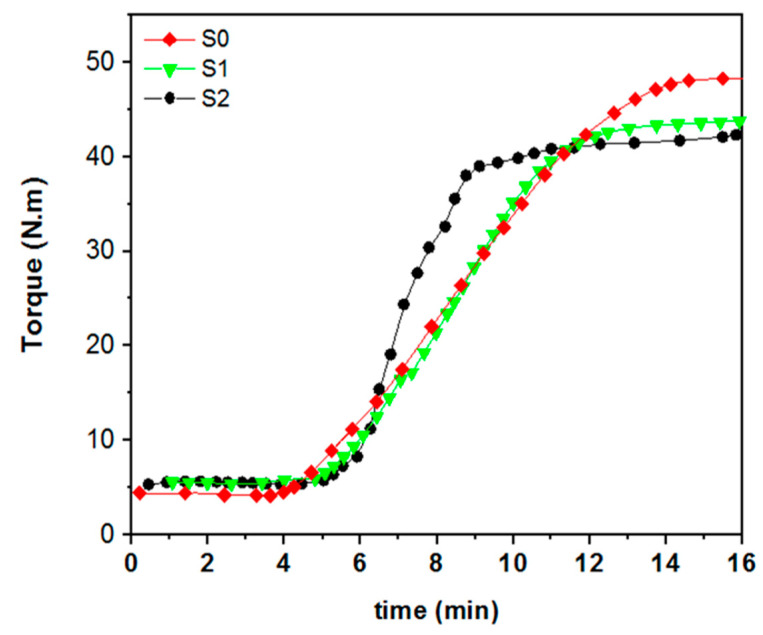
Rheometric curves of NR/CNT/GNS hybrid nanocomposite foams at 155 °C.

Controlling NR/CNT/GNS nanocomposite foams’ curing process is essential to fabricate foams with specific properties and morphologies. The effect of the nanofiller’s content on the curing behavior of rubber foams at 155 °C is displayed in Figure 1. It can be seen that increasing the CNT and GNS content accelerates the curing process by decreasing the scorch time (*t_s_*, the time that the curing process starts), the curing time (*t*_90_, when the curing process achieves 90%), the final torque or the torque of cured compounds (*M_H_*), and the delta torque or the torque difference between the cured and uncured compound (Δ*M* = *M_H_* − *M_I_*). However, it increased the curing rate (*CRI* = $\frac{100}{t_{90}\cdot t_{s}}$) [25], the initial torque, or the torque of the uncured compounds (*M_I_*). *M*_90_ can be calculated using Equation (1) [26] as follows:

*M*_90_ = *M_I_* + 0.9 (*M_H_* − *M_I_*) (1)

The result shows that the curing rate significantly increased with increasing CNT and GNS content from 0 to 2 phr, while decreasing the scorch time and the cure time from 6.81 to 5.08 min and from 14.3 to 11.12 min, respectively. This could be related to the fact that CNT/GNS hybrid nanofiller can act as accelerators to the curing process, which is in agreement with what was observed for each nanofiller individually [27,28]. In addition, increasing the CRI from 13.34 (in the S0 sample) to 16.55 (in the S2 sample) showed that the cure rate had tremendously increased by increasing CNT/GNS content. The higher cure rate of S2 in comparison to S0 confirmed the accelerating effect of hybrid nanofiller on cure reaction of NR foam. As demonstrated in Table 2, the initial torque (*M_I_*) increased with nanofiller loading, which could be due to the increase in viscosity as a result of the presence of solid materials in the polymer matrix, which limits the chains’ mobility by creating physical interactions with NR chains. Restricted viscous motions of polymer chains’ segments in presence of fillers and reinforcement were reported in the literature [29]. Additionally, adding higher amounts of the physical hybrids of CNT to GNS limits the polymer chains in the sintering reaction and reduces the amount of lattice created. Such a decrease in the percentage of networks created can be seen in reducing *M_H_* (Table 2) by adding higher amounts of nanohybrids.

### 3.2. Morphology

**Figure 2:**
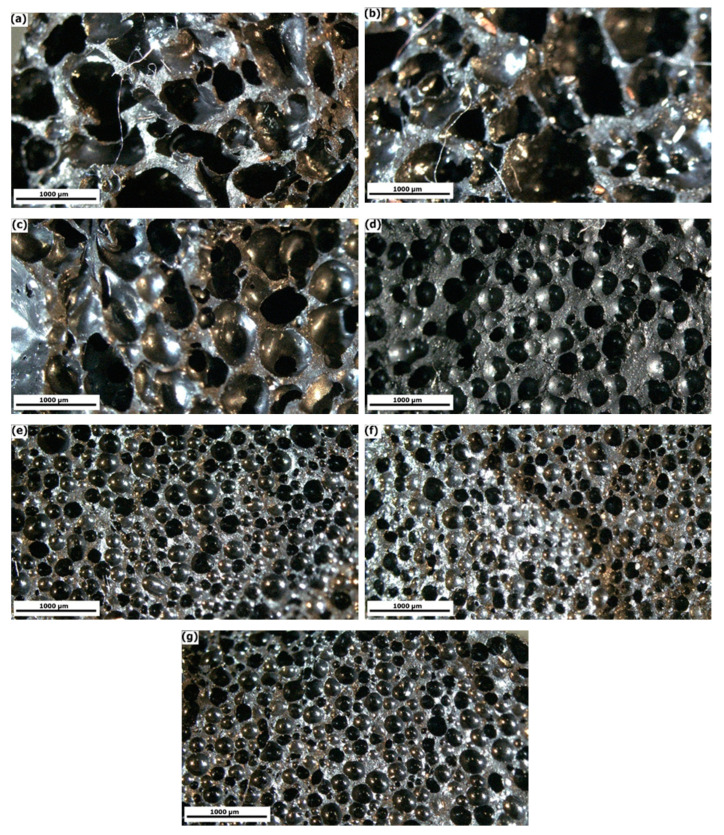
Cell morphology of NR/CNT/GNS nanocomposite foams with different CNT/GNS hybrid content: (**a**) 0 phr, (**b**) 0.1 phr, (**c**) 0.25 phr, (**d**) 0.5 phr, (**e**) 1 phr, (**f**) 1.5 phr, and (**g**) 2 phr.

To investigate the effect of the CNT/GNS hybrid on the cell structure of the NR nanocomposite foam samples, the morphology of the samples was studied using an optical microscope. Figure 2 displays images of the surface of NR/CNT/GNS foam nanocomposite samples. The qualitative investigation by optical microscopy showed that increasing the CNT/GNS hybrid content increased cell number and cell density while decreasing cell size and cell wall thickness. These changes in cell structure can be interpreted by the competition between the pressure of the released blowing agent and the rubber module that resists the growth of the bubble. The former is known as the driving force of the foaming process and the latter plays the role of inhibiting the force of the foaming process [30]. While these cavities have become more spherical in shape and closer to each other with the addition of CNT/GNS hybrid nanoparticles, they became more uniform overall. These results could be related to both the nucleation and the viscosity effect of the nanoparticles, and it is likely that the dispersed nanoparticles may act as nucleating sites that facilitate the bubbling process, consequently leading to smaller cells [30].

**Figure 3:**
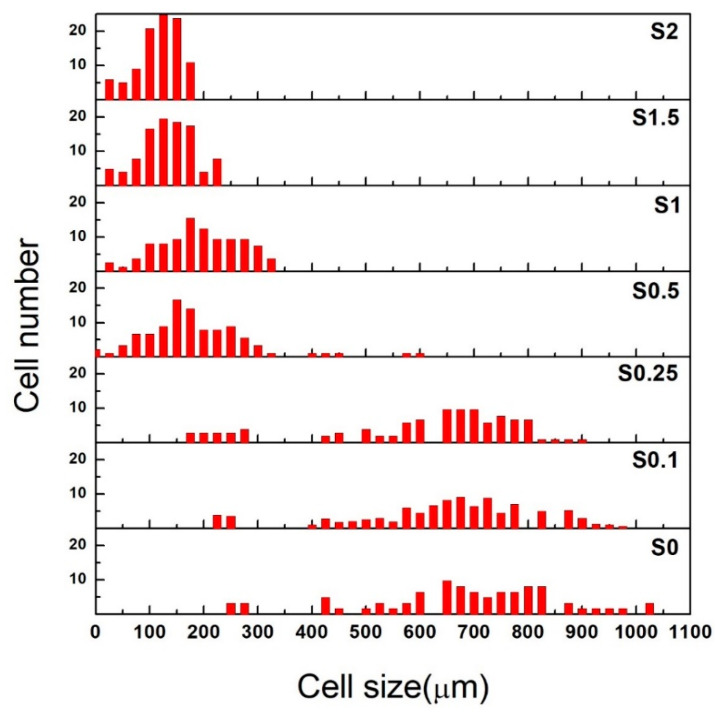
Cell size distribution of NR/CNT/GNS nanocomposite foams.

Figure 3 shows the quantitative result of the measured inner cell diameter (or the cell size) of the NR/CNT/GNS foams prepared with various hybrid nanofiller contents as histograms. The cell diameters ranged from 200 to 1000 μm, depending on CNT/GNS loading. The cell size distribution curve’s peak in the S0 sample was at 610 μm, but it shifted toward lower values with an increase in the CNT/GNS hybrid content (i.e., 110 μm for the S2 sample). Moreover, the cell size distribution of the S20 sample had a much narrower distribution compared to the S0 sample. The distribution of the NR/CNT/GNS foam cells’ inner diameters was quantified by calculating the polydispersity index (PDI), based on Equation (2) [31]:

(2) $$\mathrm{PDI}=\frac{D_{w}}{D_{n}}$$

where Dn=∑ni·Di∑ni and Dw=∑ni·Di2∑ni·Di are the number and the weighted average of the cell size, respectively.

The PDI value close to unity indicates a very uniform cell size distribution [31], which is essential for developing a product with isotropic mechanical properties. Figure 3 shows that greater uniformity in the cellular structure of the NR/CNT/GNS foams can be obtained by increasing CNT/GNS hybrid content. As previously noted, the NR foam compound’s initial modulus and cure rate increased by adding higher amounts of CNT/GNS hybrid nanofiller. In other words, the inhibiting force, which resists the foam cells’ expansion, has increased by increasing the hybrid nanofiller loading, while the total amount of expansion is the same. Thus, the average cell size decreased, and the cell size distribution became narrower.

**Figure 4:**
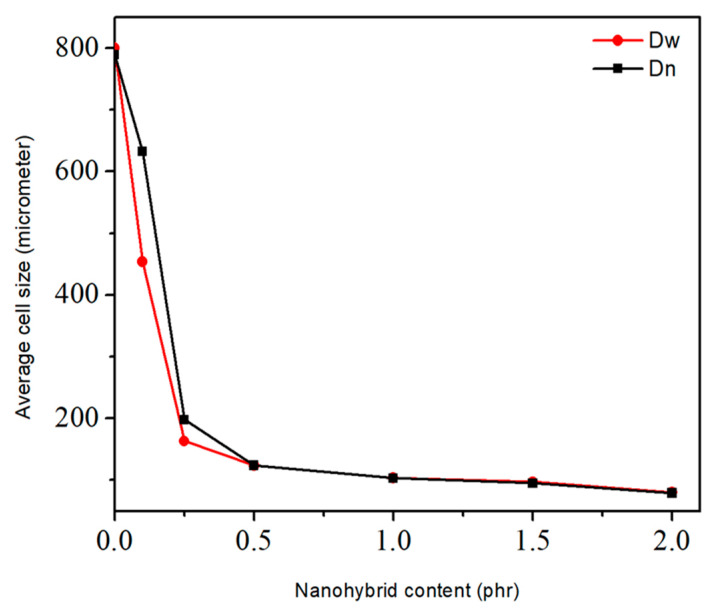
Variation of average cell size of NR/CNT/GNS foam nanocomposites versus. CNT/GNS hybrid content.

Figure 4 shows the relationship between the hybrid particle content and the average cell size. Additionally, t a significant reduction in the average cell size in NR foams reinforced by hybrid nanoparticles was observed. As mentioned, these results could be related to both the nucleation and the viscosity boosting effect of the nanoparticles. Overall, it was clear that with the addition of the hybrid nanoparticles, the cell size gradually decreased from 800 μm to 80 μm.

### 3.3. Hardness and Resilience

**Figure 5:**
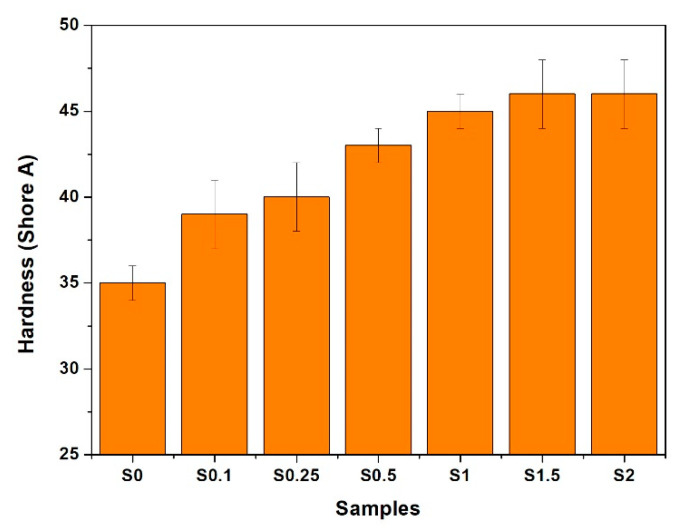
The variation in hardness in NR foams containing different content of GNS/CNT hybrid.

**Figure 6:**
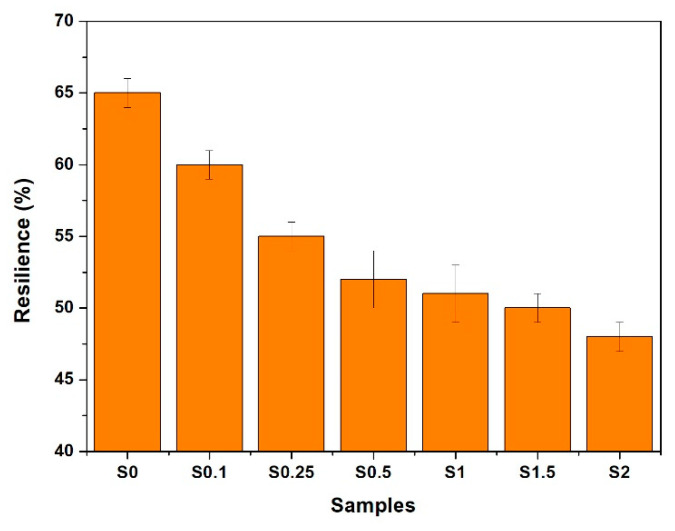
The variation in resilience in NR foams containing different content of GNS/CNT hybrid.

Hardness and resilience tests were used as a criterion to determine the elastic properties of NR/CNT/GNS rubber foams, and the data are illustrated in Figure 5 and Figure 6, respectively. As shown in Figure 5, by adding GNSs and CNTs to the NR matrix, a significant change in hardness was observed with low filler loading levels (0.5–1 phr). However, the hardness stabilized and did not display statistically significant change beyond 1 phr loading. 

A correlation between the hardness of the foams was observed with the increase in the hybrid filler loading levels, while the correlation between the resilience and the hybrid filler content was inverse. For instance, adding only 0.1 phr of CNT/GNS to the NR foam increased the hardness from 35 to 39 Shore A and decreased the resilience from 65% to 60%, while adding 2 phr of CNT and GNS had no effect on the hardness while substantially reducing the resilience (48−50%). Increasing the CNT/GNS content decreased the resilience because of the strong filler–rubber interaction, higher crosslink density, and lower NR chain flexibility. On the other hand, hardness could increase via either or both mechanisms: hindering the rubber chain mobility caused by higher curing density and filler–rubber interactions and reducing the average foam cell size (discussed above). Similar behavior was observed for polymer solid or foam composites reinforced by other types of nano reinforcements [18,30].

### 3.4. Compression Test

**Figure 7:**
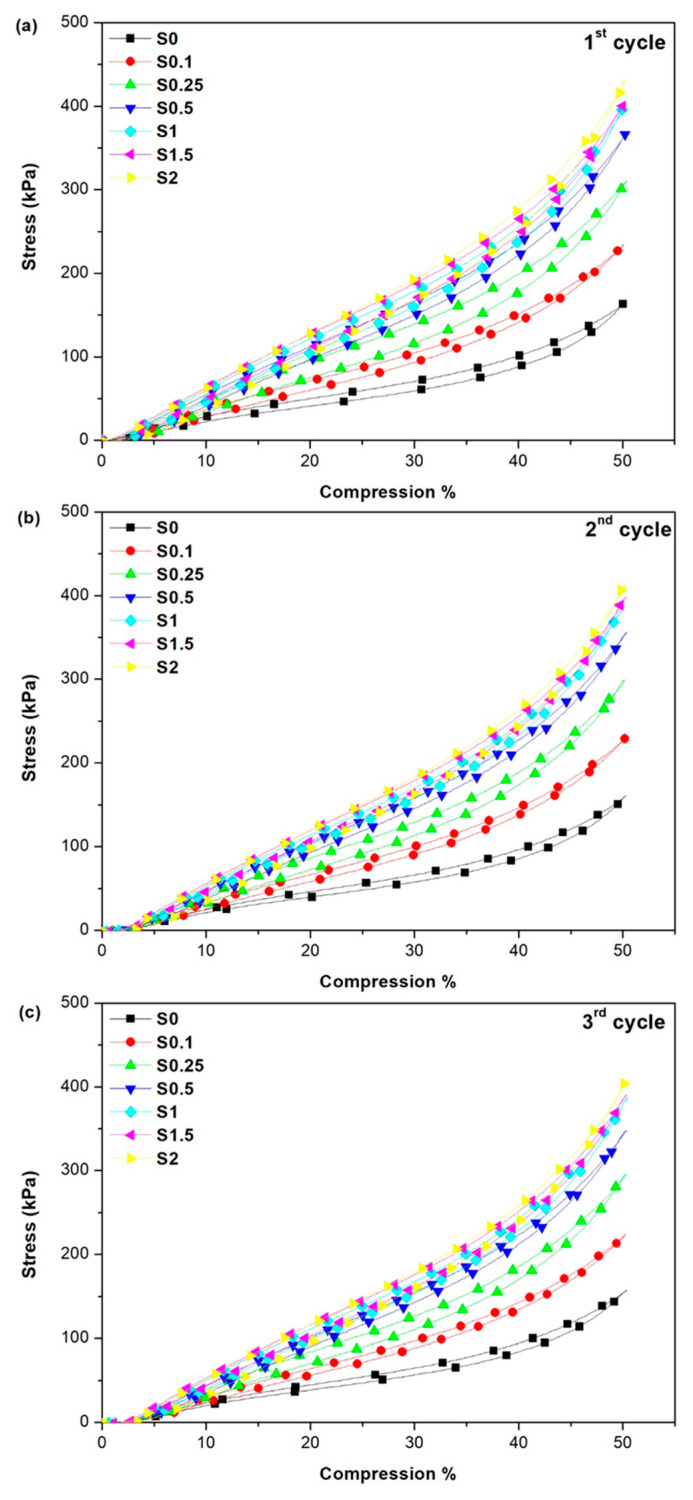
The effect of CNT/GNS hybrid content on the stress−strain behavior of the NR/CNT/GNS foams: (**a**) first cycle, (**b**) second cycle, and (**c**) third cycle.

The introduction of nano reinforcement leads to a noticeable change in the mechanical behavior of elastomeric materials under cyclic force. Figure 7 shows the effect of CNT/GNS hybrid content on the stress–strain behavior of the NR/CNT/GNS foams during a cyclic compression test. We found that the modulus and strength (i.e., stress at 50% strain) of the foams increased steadily with increasing CNT/GNS hybrid content in the NR/CNT/GNS foams. The extremely high mechanical strength of the individual CNTs provides superstrong engineering materials that can sustain stress perfectly. It is well established that nanomaterials have a high reinforcing effect in increasing the modulus of polymer foams [18]. The rubber foams can be considered as a composite of NR/CNT/GNS matrix with a dispersed gas cell phase. Therefore, the foam’s compressive stress–strain modulus depends on the mechanical properties of both individual phases and morphology. On the other hand, the moduli of the foams all increased because the interconnected 3-D framework of the CNT/GNS hybrid can effectively resist deformation and withstand force. The formation of the 3-D network of CNT/GNS in a solid polymer matrix has been reported before [24,32]. In addition, the increase in the modulus was expected due to the hardening effect of the CNT/GNS hybrid as a consequence of its stiff nature. As can be noted from Figure 7, increasing the nanofillers’ content from 0 to 2 phr improved the compression behavior and increased the stress at 50% strain from 150 kPa to 440 kPa, which are related to higher modulus, smaller cell size, and robust matrices. Thus, an increased CNT/GNS hybrid content can increase the modulus of the foam via the two possible mechanisms; the matrices’ modulus increased due to the reinforcing effect of the nanofillers caused by a 3-D network of inherently stiff nanomaterial, and reduction of the foam’s cell size.

Fatigue behavior is associated with a decrease in strength under cyclic loads, localized and progressive damage to the polymer structure due to cyclic loading. This may cause fractures or incomplete cracks after a sufficient number of compressive cycles. The S2 sample displayed the best cyclic stability, which indicates that the CNT/GNS hybrid can change the dynamic molecular properties of NR and enhances its resistance to large deformation due to repeated stress. Moreover, the nanofillers could act as nanobarriers to crack propagation, thereby increasing matrix fatigue resistance. 

The evolution of the stress–strain hysteresis loop of each NR/CNT/GNS nanocomposite foam samples with various content of CNT/GNS is provided in the supporting information (Figures S1 to S7) for better comparison. The significant difference in the width of the loops in Figures S1 to S7 proves the important role of the hybrid filler content as a parameter to control rubber deformation and ratcheting progress over stress cycles. Over the first cycle, the rubber blend S0 sample with no filler content presented a wide hysteresis loop indicative of dramatic plastic deformation. The hysteresis loop in samples containing a higher amount of CNT/GNS became narrower than that of the pristine foam. This means that as the magnitude of CNT/GNS content increases, stress–strain hysteresis loops gain higher stiffness and modulus of elasticity. By progressing loading cycles, the area within the individual hysteresis loops gradually drops, indicating decay in ratcheting rate in composite samples. The width of the loop or the area between the loading and unloading cycle corresponds to dissipated energy in tested samples over the loading/unloading cycles leading to heat build-up in specimens [33]. Thus, the narrower hysteresis of the foam samples with higher content of CNT/GNS hybrid nanofiller revealed their lower ability to dissipate the exerted energy which is in agreement with mechanical results observed above.

### 3.5. Thermal Degradation

**Figure 8:**
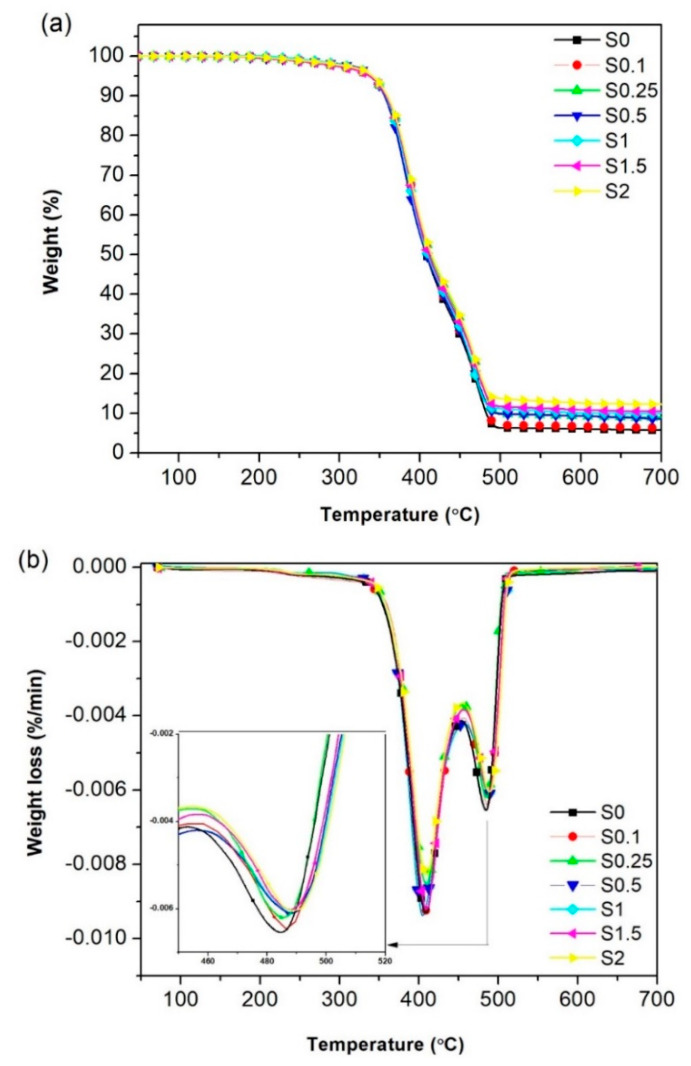
Thermal degradation behavior of the NR/CNT/GNS foams with different hybrid nanofiller content: (**a**) TGA curve and (**b**) DTG curve.

The thermo-oxidative stability results of NR/CNT/GNS foams obtained from the TGA and its derivative differential thermogravimetry (DTG) are shown in Figure 8. It can be seen that all the samples have a two-step thermal degradation in TGA curves (Figure 8a). Additionally, the DTG plots in Figure 8b showed two distinct peaks that correspond to the local maximum rates of weight loss in all nanofillers’ contents. The first step around 400 °C was related to the decomposition of small molecules and additives such as oil, blowing agent, etc. [34]. The second degradation step occurred in the region of 450−500 °C and was related to the NR degradation. In Table 2, the thermal properties of the prepared foams are directly compared to highlight the effect of CNT/GNS hybrid nanofiller on the degradation of the NR foams. It was observed that the degradation temperatures of nanocomposites at 5% and 50% weight loss are higher than that of raw rubber and with increasing hybrid nanofiller content. The percentage of remained ash rose from 5.7% to 12.23% by weight. A significant temperature rise was also observed at 50% and 5% weight loss, which increased from 407 °C to 414 °C and from 339 °C to 346 °C, respectively. This increase could be related to the interaction between NR and the nanomaterials, limiting the release of gases from the heat degradation as it causes them to cross a more tortuous path. The increase in the thermal stability of polymer composites having well-dispersed nanofiller has been reported in the literature [35].

The maximum DTG peaks in the samples were elevated by increasing the nanofillers’ content in both weight loss steps. The addition of inorganic fillers such as the GNS/CNT hybrid causes higher thermal resistance in the rubber due to their higher inherent thermal resistance compared to the NR. Therefore, the thermal stability of the nanocomposites increases when the CNT and GNS are incorporated, and these nanomaterials can act as a delaying agent in the degradation of rubber matrices. This can be ascribed to the ability of the carbon-based fillers that contribute to thermally protect the polymer matrix and hinder the transport of degradation volatiles through the matrix by the formation of a tortuous path [36]. The weight of the sample at the first T_Peak_ decreased slightly from 50.54% to 50.18%; however, a significant rise in the weight of the samples was observed at the second T_peak_, which increased dramatically from 6.77% to 14.28%.

### 3.6. Dynamical Mechanical Analysis (DMTA)

DMTA is a technique used to analyze and characterize foam nanocomposite samples based on their response to an applied dynamical, mechanical, and thermal force. DMTA results are often expressed in terms of the storage modulus (E’), loss modulus (E’’), and loss factor (tan δ), which describe the ability of the materials to lose energy as heat to mean damping and their ability to revert to their original shape and size from the deformation when the oscillating applied force is removed.

**Figure 9:**
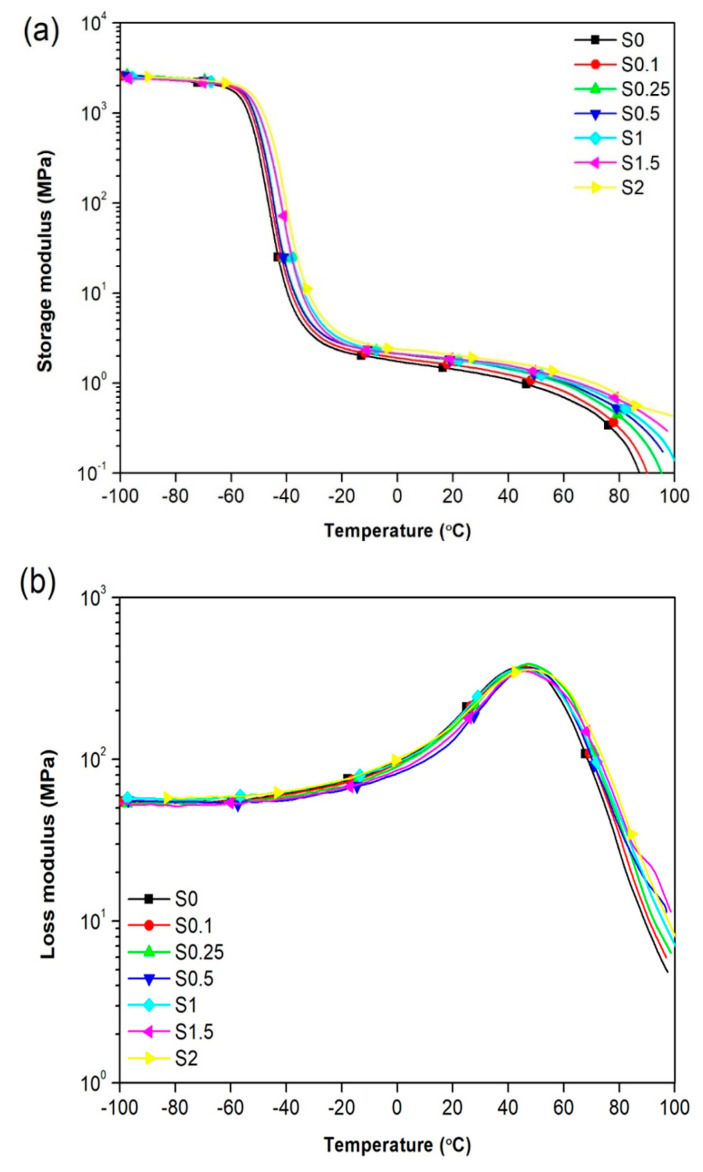

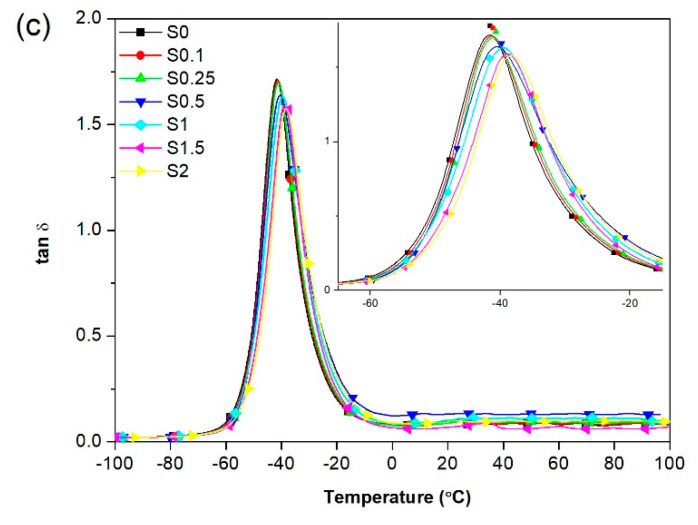
Dynamic properties of NR/CNT/GNS nanocomposite foams as a function of temperature: (**a**) variation of storage modulus (E’), (**b**) variation of loss modulus (E’’), and (**c**) variation of dissipation peak (tan δ).

The E’ test data used to describe the stiffness of composite materials and their variation as a function of temperature is presented in Figure 9a for all foam nanocomposite samples. The observed increase in E’ values above T_g_ with increased GNS/CNT content can be attributed to increasing GNS/CNT hybrid nanofiller concentration. It should be noted that this increase in E’ is negligible at temperatures below the glass transition (T_g_) due to the rigidity of the nanomaterials. As the temperature rose from −100 °C to −50 °C, the E’ values decreased for all samples. A substantial decrease in E’ was seen in the temperature range between −60 °C and −40 °C of the curve related to the matrix’s glass transition temperature. The observed increase in E’ values above T_g_ can be attributed to the interaction between the polymer matrix (NR) and the CNT/GNS hybrid nanofillers that led to the reinforcement phenomenon. Strong interactions preserve the porous structure against temperature rise [37]. E’’ is a material’s response by losing energy to the surroundings due to the applied external oscillating force. Figure 9b indicates the variation of E’’ for NR/CNT/GNS nanocomposite foams as a function of temperatures. It was noted that the E’’ curve of NR/CNT/GNS containing a higher amount of CNT/GNS hybrid nanofiller showed a slightly wider peak in the E’’ curve, which suggests a broad relaxation time distribution of NR chains. It is well-known that the broadness of the peak in multicomponent polymeric systems is primarily controlled by heterogeneity: either the intrinsic differences among components’ relaxation properties (dynamic heterogeneity) or their detailed state of local concentration fluctuations (local heterogeneity) [38]. The dispersion of rigid nanoparticles in polymeric matrices resulting in local and dynamic heterogeneity can lead to significant damping capability, which is mutually correlated with a broader peak of E’’. 

Tan δ, also called the loss factor, is calculated from the ratio of loss modulus to storage modulus (E’’/E’). The tan δ curves are plotted as a function of temperature for all samples, as shown in Figure 9c, and the data are further tabulated in Table 3. Comparing the maximum T_g_ values of the samples provided in Table 4, the T_g_ increased from 42.14 °C to 52.29 °C. It is observed in Figure 9 and that Table 3 peak of this curve (tan δ_max_) decreased with increasing CNT and GNS amount. The results show that the glass transition temperature of the S0 samples increased slowly from −45.77 °C for the S2 sample to −38.69 °C. This could be due to the physical interactions between the nanofillers and the rubber matrix. Figure 9c shows that the T_g_ of the nanocomposite had a slight transition to higher temperatures. This increase in T_g_ could be correlated to a restriction of the mobility of the segments in the matrix in the presence of carbon-based nanofillers [39,40]. 

## 4. Conclusions

In this study, the effect of incorporating GNS/CNT on the curing behavior, the foam morphology, and the mechanical and thermal properties of the NR foams was investigated. The curing characteristics showed that the addition of a hybrid nanofiller in the NR reduced the cure start time (T_s_) and sped up the overall curing reaction by limiting the polymer chains’ mobility. It was also observed that the GNS/CNT incorporation resulted in greater hardness and lower resilience of the NR composites, which was attributed to the good interaction between the polymer matrix and the physical hybrid of CNT/GNS. The compression test results showed that the addition of the nanomaterials increased the compressive strength of all formulations. The CNT/GNS hybrid nanofillers provided the natural rubber with better resistance to large deformation when exposed to repeated stress cycles (fatigue). The DMTA results revealed that the addition of CNT/GNS nanohybrid into the natural rubber matrix provided higher storage modulus in the foam samples due to the nanohybrid material’s inherent high hardness and modulus. It was also shown that the peak of the loss factor in nanocomposites decreased with increasing nanofiller content and slightly increased the glass transition temperature (T_g_). The TGA study indicated that the addition of GNS/CNT hybrid caused higher thermal stability in the NR foams by acting as a delaying agent in the degradation of the rubber matrix. With the increase in CNT/GNS content, the residual weight increased by up to 10%. The morphological results from optical microscopy also illustrated that increasing the CNT/GNS hybrid nanofiller content produced more cells with smaller cell sizes and cell wall thickness. Samples with lower nanofiller content have elongated and irregular cells. Contrarily, formulations with higher GNS/CNT hybrid loading levels resulted in cells that were more spherical and closer in size to each other with an overall improved uniformity and narrower distribution.

## Figures and Tables

**Table 1:** Formulation of NR/CNT/GNS nanocomposite foams.

Ingredients												Sample Code
Master Batch	ADC	Oil	Sulfur	ZnO	Stearic Acid	MBTS	CBS	CB	GNS	CNT	NR	
0	6	5	1.5	4.5	2	0.6	0.6	10	0	0	100	S0
0.771	6	5	1.5	4.5	2	0.6	0.6	10	0.1	0.1	100	S0.1
1.928	6	5	1.5	4.5	2	0.6	0.6	10	0.25	0.25	100	S0.25
3.857	6	5	1.5	4.5	2	0.6	0.6	10	0.5	0.5	100	S0.5
7.714	6	5	1.5	4.5	2	0.6	0.6	10	1	1	100	S1
11.571	6	5	1.5	4.5	2	0.6	0.6	10	1.5	1.5	100	S1.5
15.428	6	5	1.5	4.5	2	0.6	0.6	10	2	2	100	S2

**Table 2:** Curing characteristics of NR/CNT/GNS hybrid nanocomposite foams at 155 °C.

Sample Code	*M_L_* (N·m)	*M_H_* (N·m)	*M_90_* (N·m)	*ΔM* (N·m)	*T_s_* (min)	*T_90_* (min)	*CRI* (%/min)
S0	4.68	48.1	43.76	43.42	6.81	14.3	13.35
S0.1	4.8	47.78	43.48	42.98	6.19	14.0	12.80
S0.25	5.01	48.31	43.98	43.3	6.08	13.9	12.78
S0.5	5.2	43.2	39.41	38	5.89	13.7	12.80
S1	5.34	43.5	39.68	38.16	5.63	12.5	14.55
S1.5	5.50	43.8	39.97	38.3	5.43	11.7	15.94
S2	5.46	42.5	38.79	37.04	5.08	11.12	16.55

**Table 3:** Thermal degradation characteristics of the NR/CNT/GNS Foam.

Samples	1st Step of Weight Loss		2nd Step of Weight Loss		T_5_ (°C)	T_50_ (°C)	Ash (%)
	T_peak1_ (°C)	W_1_ (%)	T_peak2_ (°C)	W_2_ (%)			
S0	405.14	50.54	485.11	6.77	339	407	5.7
S0.1	405.88	50.43	486.07	8.31	339	407	6.3
S0.25	408.55	50.78	485.98	9.92	340	408	8.50
S0.5	409.15	50.23	487.59	10.76	340	409	8.49
S1	410.15	50.68	487.58	11.93	341	411	10.03
S1.5	410.25	49.89	489.01	12.65	344	413	10.43
S2	410.87	50.18	489.32	14.28	346	414	12.23

T_Peak_: Maximum temperature of the DTG peak; W: Weight of the sample at T_Peak_; T_5_: Temperature at 5% of weight loss; T_50_: Temperature at 50% of weight loss.

**Table 4:** Variation in T_g_ (Measured from the Peak of the tan δ Curve).

T_g_ (°C)	Intensity of tanδ Peak at T_g_	Sample Code
42.14	1.92	S0
45.66	1.90	S0.1
47.09	1.89	S0.25
47.78	1.82	S0.5
48.68	1.81	S1
50.48	1.77	S1.5
52.29	1.75	S2

## Data Availability

Not applicable.

## Supplementary Materials

The following are available online at https://www.mdpi.com/article/10.3390/polym13142346/s1. Figure S1. Evolution of stress-strain hysteresis loops for NR nanocomposite foam samples with 0 phr of physical hybrid of CNT/GNS at different cycles. Figure S2. Evolution of stress-strain hysteresis loops for NR nanocomposite foam samples with 0.1 phr of physical hybrid of CNT/GNS at different cycles. Figure S3. Evolution of stress-strain hysteresis loops for NR nanocomposite foam samples with 0.25 phr of physical hybrid of CNT/GNS at different cycles. Figure S4. Evolution of stress-strain hysteresis loops for NR nanocomposite foam samples with 0.5 phr of physical hybrid of CNT/GNS at different cycles. Figure S5. Evolution of stress-strain hysteresis loops for NR nanocomposite foam samples with 1 phr of physical hybrid of CNT/GNS at different cycles. Figure S6. Evolution of stress-strain hysteresis loops for NR nanocomposite foam samples with 1.5 phr of physical hybrid of CNT/GNS at different cycles. Figure S7. Evolution of stress-strain hysteresis loops for NR nanocomposite foam samples with 2 phr of physical hybrid of CNT/GNS at different cycles.
