# Supplementary material for: Physical Hybrid of Nanographene/Carbon Nanotubes as Reinforcing Agents of NR-Based Rubber Foam
Source: Polymers (Basel). 2021 Jul 17;13(14):2346. doi: 10.3390/polym13142346 (PMC8309651; doi:10.3390/polym13142346)
Supplement: Supplementary file 1 [file polymers-13-02346-s001.zip › polymers-1276384-supplementary.pdf]

polymers

MDPI

*Supporting Information*

# Physical Hybrid of Nanographene/Carbon Nanotubes as Reinforcement of NR-Based Rubber Foam

**Sahar Shojaie [1], Ali Vahidifar [1], Ghasem Naderi [2], Elham Shokri [1], Tizazu H. Mekonnen [3] and Elnaz Esmizadeh [4,*]**

1 Faculty of Engineering, Department of Polymer Science and Engineering, University of Bonab, Bonab, Iran
2 Department of polymer processing, Iran Polymer and Petrochemical Institute, 14965/115, Tehran, Iran
3 Department of Chemical Engineering, University of Waterloo, Waterloo, ON, N2L 3G1, Canada
4 Construction Research Center, National Research Council Canada, 1200 Montreal Rd., Ottawa, ON, K1A 0R6, Canada
* Correspondence: Elnaz.Esmizadeh@nrc-cnrc.gc.ca

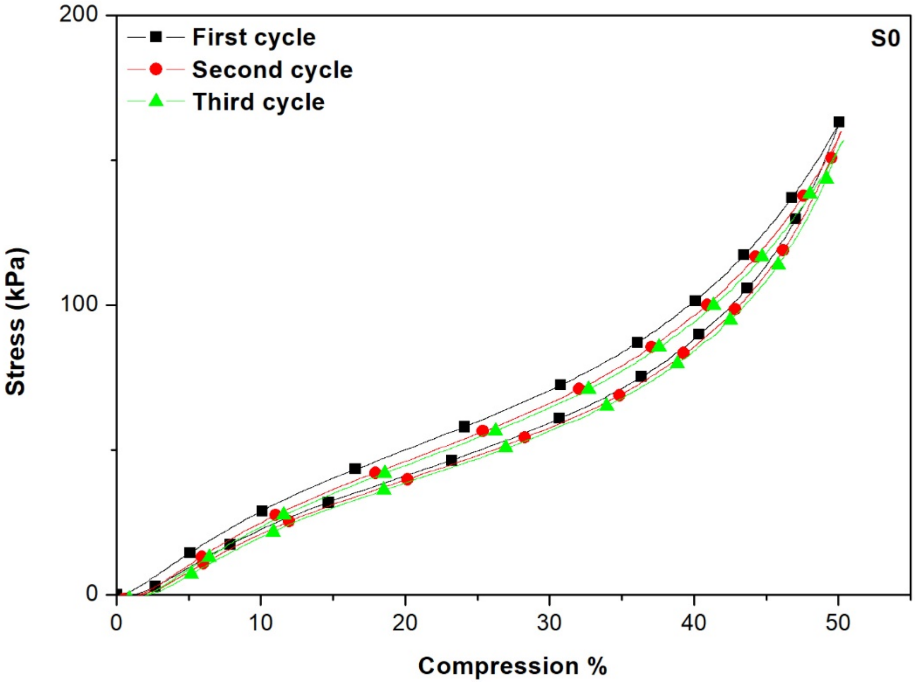


**Figure S1.** Evolution of stress-strain hysteresis loops for NR nanocomposite foam samples with 0 phr of physical hybrid of CNT/GNS at different cycles.

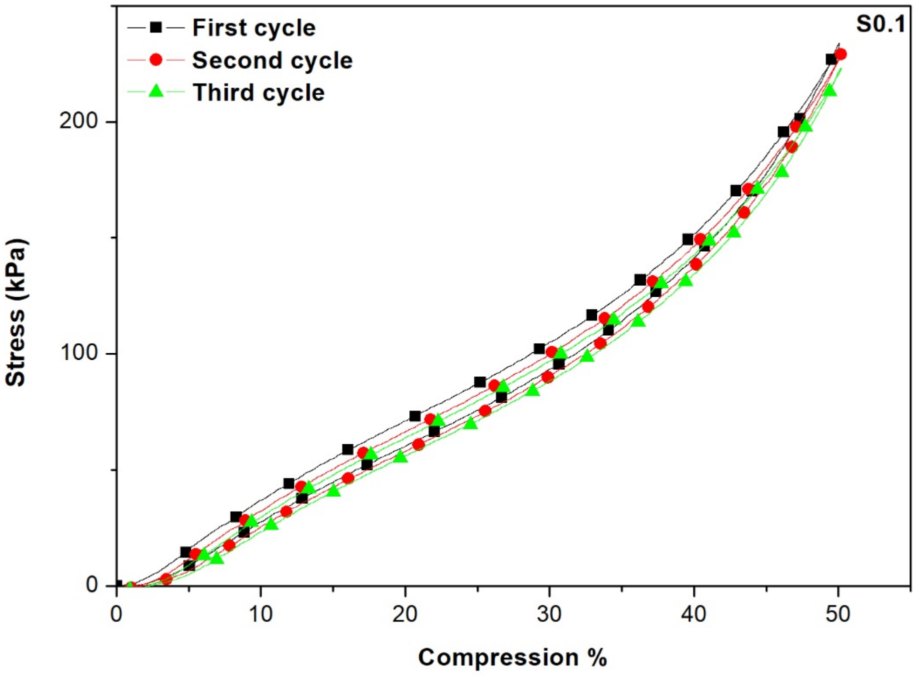


**Figure S2.** Evolution of stress-strain hysteresis loops for NR nanocomposite foam samples with 0.1 phr of physical hybrid of CNT/GNS at different cycles.

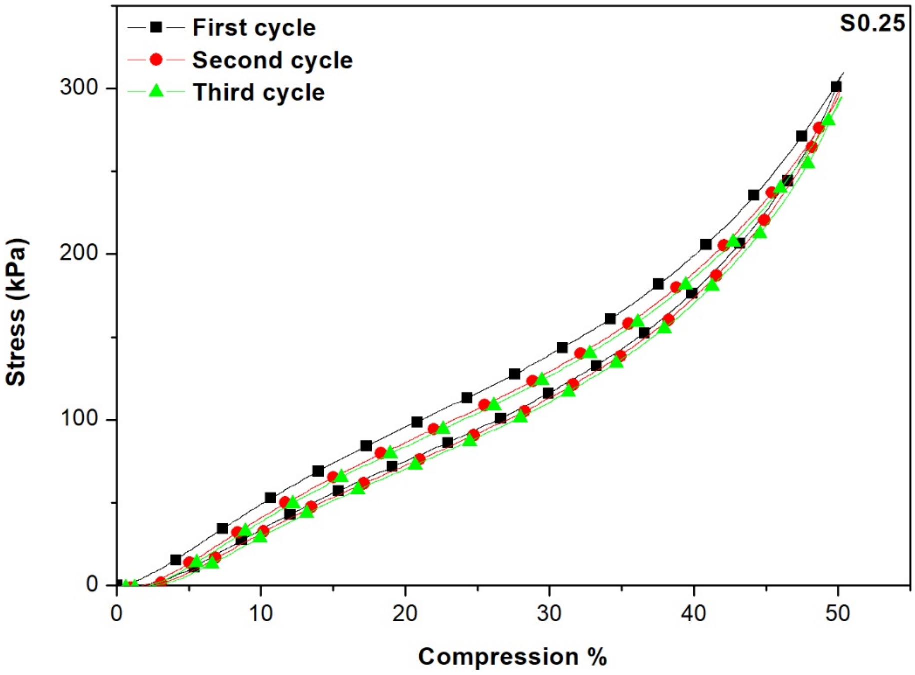


**Figure S3.** Evolution of stress-strain hysteresis loops for NR nanocomposite foam samples with 0.25 phr of physical hybrid of CNT/GNS at different cycles.

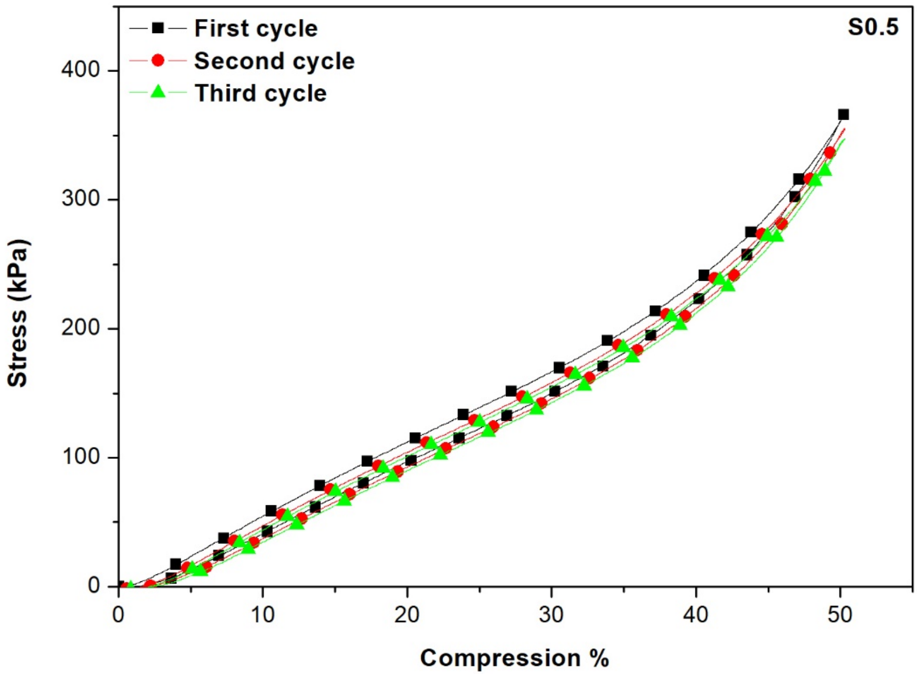


**Figure S4.** Evolution of stress-strain hysteresis loops for NR nanocomposite foam samples with 0.5 phr of physical hybrid of CNT/GNS at different cycles.

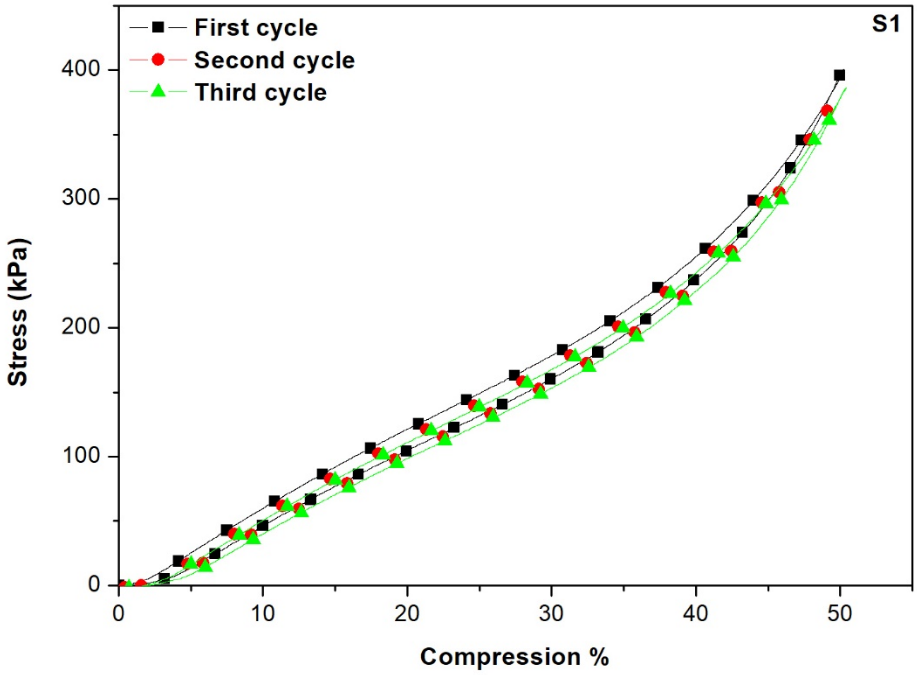


**Figure S5.** Evolution of stress-strain hysteresis loops for NR nanocomposite foam samples with 1 phr of physical hybrid of CNT/GNS at different cycles.

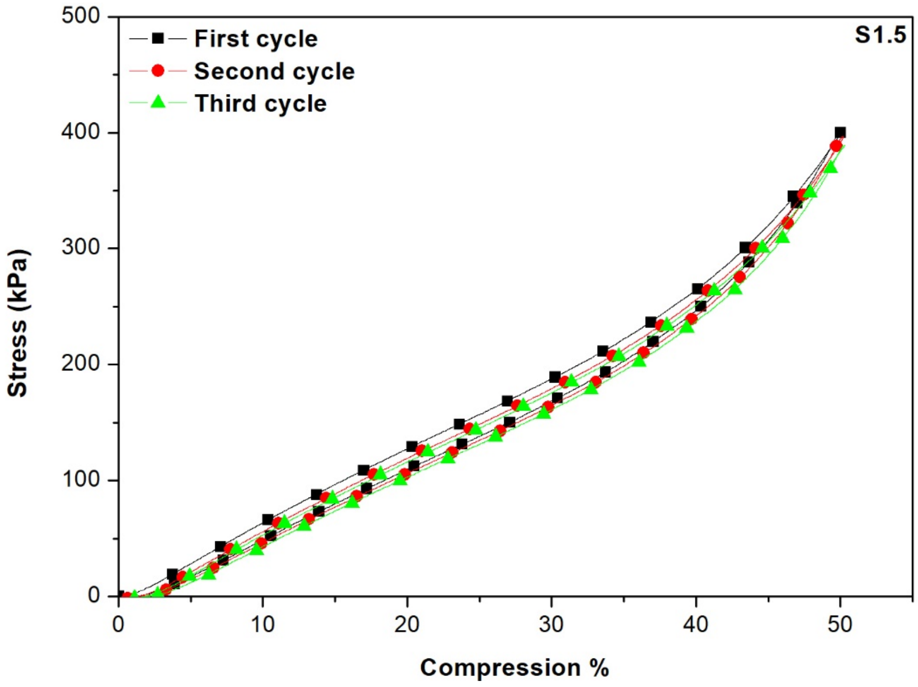


**Figure S6.** Evolution of stress-strain hysteresis loops for NR nanocomposite foam samples with 1.5 phr of physical hybrid of CNT/GNS at different cycles.

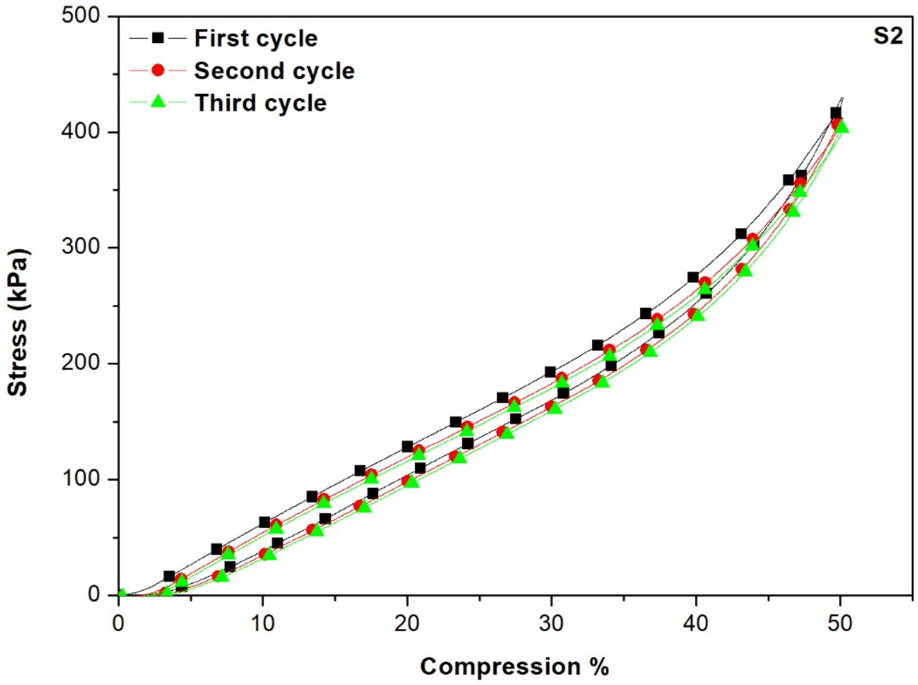


**Figure S7.** Evolution of stress-strain hysteresis loops for NR nanocomposite foam samples with 2 phr of physical hybrid of CNT/GNS at different cycles.
